# The role of FGF2 in spinal cord trauma and regeneration research

**DOI:** 10.1002/brb3.207

**Published:** 2014-01-13

**Authors:** Nimer Adeeb, Martin M Mortazavi

**Affiliations:** Department of Neurological Surgery, University of WashingtonSeattle, Washington

Please note that an article related to this editorial, “Fgf2 improves functional recovery–decreasing gliosis and increasing radial glia and neural progenitor cells after spinal cord injury,” doi: 10.1002/brb3.172, can be found here, also published in Brain and Behavior.

We read with interest the article by Goldshmit et al. in this issue of *Brain and Behavior*. They hypothesized that fibroblast growth factor 2 (FGF2), given subcutaneously in a hemisection spinal cord injury (SCI) model in mice, decreases inflammation and gliosis, increases radial glia, neural progenitor cells, neuronal survival and axonogenesis, and ultimately leads to improved functional recovery.

SCI in human affects a large group of relatively young people with many years of expected survival and severe morbidities. SCI and regeneration has been one of the major areas of research in the last decade and a lot of knowledge has been gained. Crucial to why central nervous system (CNS) does not repair itself compared to the peripheral nervous system (PNS) is the difference in the inherent abilities of the glial cells in these systems. Research has shown that the injured neurons initiate neurite outgrowth both in the CNS and PNS. In the PNS, this neurite outgrowth continues. In CNS, however, it stops for several reasons. Most important are the neurite inhibitory effect of the exposed Nogo's on the surface of the injured oligodendrocytes, the relative lack of enhanced growth factor production by injured glia in the injured area, and the cavitation and the scar tissue formation induced by the inflammatory reaction (Steward et al. 1999; Norenberg et al. 2004; Profyris et al. 2004). There is a distinct difference in production and availability of growth factors in CNS for multiple reasons. Part of SCI-research has therefore come to focus on growth factors as medical “primers” of the injured spinal cord.

There are a number of growth factors that have been shown to alter different cell types and functions, reducing the deleterious effects of an injury, while improving neuronal survival and regeneration. FGF2, which is present in both neurons and glial cells, has previously been reported to have multiple neural-promoting effects on the developing and the adult nervous system of mice and other mammals.

FGF2 has also been found to play an important role in inducing and regulating the proliferation of neural stem cells and precursors, promoting their survival and maintenance in vitro (Arsenijevic et al. 2001; Mudò et al. 2009). This mitogenic effect was also detected on spinal cord-derived neural precursors (Ray and Gage 1994). With proper induction and expansion, cultures of neural precursors were able to survive, proliferate, and migrate after engraftment at the site of SCI (Karimi-Abdolrezaee et al. 2006). FGF2 also plays a role in regulating the proliferative fate and differentiation of unipotent (neuronal) and bipotent (neuronal/astroglial) mouse-derived neural precursor cells, and hence, the generation of neurons and astrocytes in the developing CNS (Vescovi et al. 1993).

After administration of neutralizing antibodies against endogenous FGF2 (Tao et al. 1997), significant depression of the rate of neural proliferation and development, was seen. In mice models, FGF2 was found to reduce inflammation by decreasing multiple inflammatory cells and markers such as macrophages, microglia, CD8 T-cells (Ruffini et al. 2001; Rottlaender et al. 2011), and limited the CD44-mediated leukocyte migration (Jones et al. 2000). Contradictory results have been shown on its effect on astrocytosis and gliosis (Reilly et al. 1998; Goddard et al. 2002; Kasai et al. 2010). However, an interesting observation in zebra fish was that maturing astrocytes exhibited long bipolar processes, which bridged across the two sides of the injured spinal cord. These glial bridges were found to play a role as routes for axonal growth during regenerative neurogenesis, and its formation was dependent on the presence of FGF-signaling (Goldshmit et al. 2012). In addition to its direct effects, exogenous FGF2 also functions indirectly via activation of endogenous FGF2 in brain ischemia model in rat (Liu et al. 2006). Intrathecal administration of FGF2 after moderate or severe SCI in rats was associated with earlier and more pronounced hind limb movements and coordination compared to control group (Rabchevsky et al. 1999, 2000; Kasai et al. 2010). These remarkable effects of FGF2 have, therefore, led to its use in different treatment strategies for neural injury.

In this study, the authors reported using subcutaneous FGF2 injection as early as 30 min after injury and up to 2 weeks. This method was unique up to our knowledge in treatment of similar conditions because it has been shown that with systemic administration, FGF2 will exhibit unequal distribution to the body organs due to its heparin-binding domain (Epstein et al. 2001), and will be exposed to rapid tissue clearance (Kang et al. 2013). More importantly, it has also been reported that blood-spinal cord barrier (BSB) has a very limited permeability to FGF2 (Epstein et al. 2001; Kang et al. 2010a,b2010b). Therefore, previous studies, including both cited references by the current authors for FGF2 application in rodents with SCI (Lee et al. 1999; Rabchevsky et al. 1999), have administered FGF2 either intrathecally or directly into the site of SCI. Following brain injury, however, some studies have shown that the disrupted blood–brain barrier (BBB) becomes more permeable for some hours to days, and this window can be used for systemic infusion of FGF2 with promising results (Liu et al. 2006). Whether this time frame also represents a therapeutic window for FGF2 administration following SCI in this study is not known.

It is also important to emphasize that a hemisection model is used in this study. Although the majority of spinal cord injuries in human are contusions rather than transections, in experimental spinal cord research, a transection model is preferred, because crossover through the uninjured side, is a well-known phenomenon (Cowley et al. 2008; Oudega and Perez 2012). Transection, however, leads to animal morbidity and loss, and many times, a hemisection model is used and the crossover effect is partially compensated by having controls and shams. However, having controls and shams is not an absolute compensation and transection models are preferred.

In summary, this collection of pilot studies has focused on a number of important parameters crucial for experimental animal spinal cord research. The overall results at first sight look promising. Some of the results have been studied earlier or at least in other species but the endpoints are valid and interesting. Nevertheless, the number of animals in each of these five pilot studies is low. Not specific for this study, we should remember that many of the animal studies with promising results are not reproducible (Button et al. 2013), the hemisection model is not optimal (Cowley et al. 2008), mice have an extreme ability for functional potentiation of the uninjured neurons (Steward et al. 1999), and most importantly, the animal results, although the only way to go, are hard to be translated to human.

Given the promising data, a larger study to reproduce and confirm the results for everyone and each of the pilot studies would be desirable.

## References

[b1] Arsenijevic Y, Weiss S, Schneider B, Aebischer P (2001). Insulin-like growth factor-I is necessary for neural stem cell proliferation and demonstrates distinct actions of epidermal growth factor and fibroblast growth factor-2. J. Neurosci.

[b2] Button KS, Ioannidis JPA, Mokrysz C, Nosek BA, Flint J, Robinson ESJ (2013). Power failure: why small sample size undermines the reliability of neuroscience. Nat. Rev. Neurosci.

[b3] Cowley KC, Zaporozhets E, Schmidt BJ (2008). Propriospinal neurons are sufficient for bulbospinal transmission of the locomotor command signal in the neonatal rat spinal cord. J. Physiol.

[b4] Epstein SE, Fuchs S, Zhou YF, Baffour R, Kornowski R (2001). Therapeutic interventions for enhancing collateral development by administration of growth factors: basic principles, early results and potential hazards. Cardiovasc. Res.

[b5] Goddard DR, Berry M, Kirvell SL, Butt AM (2002). Fibroblast growth factor-2 induces astroglial and microglial reactivity in vivo. J. Anat.

[b6] Goldshmit Y, Sztal TE, Jusuf PR, Hall TE, Nguyen-Chi M, Currie PD (2012). FGF-dependent glial cell bridges facilitate spinal cord regeneration in zebrafish. J. Neurosci.

[b7] Jones M, Tussey L, Athanasou N, Jackson DG (2000). Heparan sulfate proteoglycan isoforms of the CD44 hyaluronan receptor induced in human inflammatory macrophages can function as paracrine regulators of fibroblast growth factor action. J. Biol. Chem.

[b8] Kang CE, Clarkson MR, Tator CH, Yeung IWT, Shoichet MS (2010a). Spinal cord blood flow and blood vessel permeability measured by dynamic computed tomography imaging in rats after localized delivery of fibroblast growth factor. J. Neurotrauma.

[b9] Kang CE, Tator CH, Shoichet MS (2010b). Poly(ethylene glycol) modification enhances penetration of fibroblast growth factor 2 to injured spinal cord tissue from an intrathecal delivery system. J. Controlled Release.

[b10] Kang CE, Baumann MD, Tator CH, Shoichet MS (2013). Localized and sustained delivery of fibroblast growth factor-2 from a nanoparticle-hydrogel composite for treatment of spinal cord injury. Cells Tissues Organs.

[b11] Karimi-Abdolrezaee S, Eftekharpour E, Wang J, Morshead CM, Fehlings MG (2006). Delayed transplantation of adult neural precursor cells promotes remyelination and functional neurological recovery after spinal cord injury. J. Neurosci.

[b12] Kasai M, Jikoh T, Fukumitsu H, Furukawa S (2010). FGF-2-responsive and spinal cord-resident cells improve locomotor function after spinal cord injury. J. Neurotrauma.

[b13] Lee TT, Green BA, Dietrich WD, Yezierski RP (1999). Neuroprotective effects of basic fibroblast growth factor following spinal cord contusion injury in the rat. J. Neurotrauma.

[b14] Liu Y, Lu J-B, Ye Z-R (2006). Permeability of injured blood brain barrier for exogenous bFGF and protection mechanism of bFGF in rat brain ischemia. Neuropathology.

[b15] Mudò G, Bonomo A, Frinchi V, Di Liberto M, Fuxe K, Belluardo N (2009). The FGF-2/FGFRs neurotrophic system promotes neurogenesis in the adult brain. J. Neural Transm.

[b16] Norenberg MD, Smith J, Marcillo A (2004). The pathology of human spinal cord injury: defining the problems. J. Neurotrauma.

[b17] Oudega M, Perez MA (2012). Corticospinal reorganization after spinal cord injury. J. Physiol.

[b18] Profyris C, Cheema SS, Zang D, Azari MF, Boyle K, Petratos S (2004). Degenerative and regenerative mechanisms governing spinal cord injury. Neurobiol. Dis.

[b19] Rabchevsky AG, Fugaccia I, Turner AF, Blades DA, Mattson MP, Scheff SW (1999). Basic fibroblast growth factor (bFGF) enhances tissue sparing and functional recovery following moderate spinal cord injury. J. Neurotrauma.

[b20] Rabchevsky AG, Fugaccia I, Turner AF, Blades DA, Mattson MP, Scheff SW (2000). Basic fibroblast growth factor (bFGF) enhances functional recovery following severe spinal cord injury to the rat. Exp. Neurol.

[b21] Ray J, Gage F (1994). Spinal cord neuroblasts proliferate in response to basic fibroblast growth factor. J. Neurosci.

[b22] Reilly JF, Maher PA, Kumari VG (1998). Regulation of astrocyte GFAP expression by TGF-*β*1 and FGF-2. Glia.

[b23] Rottlaender A, Villwock H, Addicks K, Kuerten S (2011). Neuroprotective role of fibroblast growth factor-2 in experimental autoimmune encephalomyelitis. Immunology.

[b24] Ruffini F, Furlan R, Poliani PL, Brambilla E, Marconi P, Bergami A (2001). Fibroblast growth factor-II gene therapy reverts the clinical course and the pathological signs of chronic experimental autoimmune encephalomyelitis in C57BL/6 mice. Gene Ther.

[b25] Steward O, Schauwecker PE, Guth L, Zhang Z, Fujiki M, Inman D (1999). Genetic approaches to neurotrauma research: opportunities and potential pitfalls of murine models. Exp. Neurol.

[b26] Tao Y, Black IB, DiCicco-Bloom E (1997). In vivo neurogenesis is inhibited by neutralizing antibodies to basic fibroblast growth factor. J. Neurobiol.

[b27] Vescovi AL, Reynolds BA, Fraser DD, Weiss S (1993). bFGF regulates the proliferative fate of unipotent (neuronal) and bipotent (neuronal/astroglial) EGF-generated CNS progenitor cells. Neuron.

